# A Precious Swindler

**Published:** 1870-04

**Authors:** 


### A PRECIOUS SWINDLER.

About one year ago we wrote to "Dr. J. B. Ogden," of New York, for his free formula for the cure of spermatorrhœa, and signed "John Brown, drawer, 179," to the letter. Our object being, to learn the Doctor's (?) style of doing business, in order that we might give him the benefit of a free *puff* in the BISTOURY. (He got it we believe, eh ?) Well, we have just received a communication from "MICHELIN & Co.," who purport to be located on Broadway, corner of Fulton street, New York, and who address us as John Brown, drawer 179." As we never wrote any other swindler but J. B. Ogden, under the above ficticious name, we at once conclude that J. B., has gone into new business—possibly the BISTOURY made it too *warm* for him in his old swindling operation. However, this "Michelin & Co.," want us to send them $10, for which they will forward us "a package of 25 Engravings," and a *premium* as a sample to exhibit for the sale of their goods, a splendid "Hunting Duplex Chronometer Watch"! Only think of it—twenty-five splendid engravings and a watch as good as gold, all for $10. Michelin & Co., want it distinctly understood that they offer this as an inducement, and *at a great loss*, too, to have us, Mr. John Brown, of drawer 179, become their only agent. Why, they assure us that we can make from $25 to $100 every blessed day of our lives, by buying and selling their splendid engravings and beautiful watches, at $10 a cart load. Gracious, what an opportunity to become suddenly and mysteriously rich! We read from Michelin & Co's circular that they are managers of "THE GREAT TRADE IMPROVEMENT ASSOCIATION." That said association is "incorporated and authorized by the State government." Now, *that's* an inducement. We suppose they really mean that the State authorities allow them to prosecute their swindling operations, without casting them into State prison, where they justly belong.

We advise everybody, including John Brown of drawer 179, not to meddle with Michelin & Co., or anybody else doing business after their style. Honest dealers never give away a hundred or so dollar's worth of goods for *ten dollars*. Let such swindlers alone!

